# Interventions to support shared decision making for hypertension: A systematic review of controlled studies

**DOI:** 10.1111/hex.12826

**Published:** 2018-09-17

**Authors:** Rachel A. Johnson, Alyson Huntley, Rachael A. Hughes, Helen Cramer, Katrina M. Turner, Ben Perkins, Gene Feder

**Affiliations:** ^1^ Centre for Academic Primary Care Bristol Medical School Bristol UK; ^2^ Population Health Sciences Bristol Medical School Bristol UK; ^3^ Present address: Conquest Hospital Hastings UK

**Keywords:** hypertension, patient participation, primary health care, shared decision making, systematic review

## Abstract

**Background:**

Hypertension (high blood pressure) is a common long‐term health condition. Patient involvement in treating and monitoring hypertension is essential. Control of hypertension improves population cardiovascular outcomes. However, for an individual, potential benefits and harms of treatment are finely balanced. Shared decision making has the potential to align decisions with the preferences and values of patients.

**Objective:**

Determine the effectiveness of interventions to support shared decision making in hypertension.

**Search strategy:**

Searches in MEDLINE, EMBASE, CINAHL, Web of Science and PsycINFO up to 30 September 2017.

**Eligibility criteria:**

Controlled studies evaluating the effects of shared decision‐making interventions for adults with hypertension compared with any comparator in any setting and reporting any outcome measures.

**Results:**

Six studies (five randomized controlled trials) in European primary care were included. Main intervention components were as follows: training for health‐care professionals, decision aids, patient coaching and a patient leaflet. Four studies, none at low risk of bias, reported a measure of shared decision making; the intervention increased shared decision making in one study. Four studies reported blood pressure between 6 months and 3 years after the intervention; there was no difference in blood pressure between intervention and control groups in any study. Lack of comparability between studies prevented meta‐analysis.

**Conclusions:**

Despite widespread calls for shared decision making to be embedded in health care, there is little evidence to inform shared decision making for hypertension, one of the most common conditions managed in primary care.

## 1 BACKGROUND

Shared decision making is a process by which clinicians and patients work together to make health‐care choices, based on clinical evidence and the patient's informed preferences.^1^ Shared decision making is viewed as an ethical imperative by health‐care professional regulatory bodies^2^ and is embedded in health policy in several countries, including the UK and the United States.^3^, ^4^ It is increasingly advocated in the care of all conditions, including chronic health‐care conditions such as hypertension (high blood pressure)^5^ Implementing shared decision making in routine care has proven challenging, and many barriers have been identified from both patient and health‐care professional perspectives.^6^, ^7^

Interventions to support shared decision making include those which prepare health‐care teams, individual clinicians or patients before consultations (e.g patient coaching interventions, decision aids, clinician or health‐care team training interventions), and those which help practitioners and patients make decisions together during consultations, notably decision aids. There is evidence from conditions other than hypertension that shared decision making can lead to more appropriate care,^8^ reduce overtreatment,^9^ improve health outcomes^10^ and may reduce health‐care treatment costs.^11^ A systematic review of interventions to support the adoption of shared decision making by health professionals^12^ was unable to draw conclusions about the most effective interventions for supporting health professionals’ adoption of shared decision making, due to the paucity of evidence. None of the studies in that review focused on people with hypertension. A recent systematic review of randomized controlled trials, including one study that did focus on hypertension management, found that people exposed to decision aids feel more knowledgeable, clearer about their values and may make choices more in line with their values.^8^

Hypertension affected 31% of the world's adult population in 2010^13^; it increases the risk of cardiovascular conditions such as strokes and heart attacks and is the leading preventable cause of premature death worldwide.^14^ Observational studies show a progressive rise in cardiovascular risk as systolic blood pressure rises above 115 mmHg.^15^ Hypertension is diagnosed when a person's blood pressure (BP) exceeds a threshold, typically 140/90 mmHg.^16^ Management is characterized by monitoring of blood pressure alongside other cardiovascular risk factors and the use of lifestyle measures, usually combined with antihypertensive drug treatment to reduce blood pressure below treatment thresholds. Optimal treatment targets vary and are the subject of vigorous debate.^17^ Treatment is typically lifelong with adjustment and, often, intensification of antihypertensive treatment over time. Hypertension control is frequently considered suboptimal, that is it fails to reach specified treatment targets.^18^

Achieving blood pressure control has the potential for improved outcomes and cost savings at the population level.^19^, ^20^ However, from an individual patient's perspective, the potential benefits are less certain. Options to reduce blood pressure include a choice of medications and lifestyle changes. Potential benefit will vary with an individual's overall cardiovascular risk, and potential disbenefits include medication side‐effects and the burden of having to take daily medication. Patients making decisions about antihypertensive drug treatment require discussions about treatment to be personalized in order for the decisions to make sense to them.^21^ Shared decision making for hypertension has the potential to address this challenge, yet it is unclear how best to support shared decision making for hypertension, and the effect of shared decision making on outcomes is unknown. Given the high prevalence of hypertension and its impact on cardiovascular risk, shared decision making for hypertension may have profound impacts at both individual and public health levels.

### 1.1 Objective

The main objective of this study was to determine the effectiveness of interventions, including but not limited to decision aids, to support shared decision making in hypertension. A second objective was to describe the outcomes that have been used to evaluate interventions supporting shared decision making for hypertension.

## 2 METHODS

The protocol for this systematic review was registered on PROSPERO (CRD42015014143).^22^

### 2.1 Search strategy

We used search strategies incorporating subject heading and text word searches focused on shared decision making and hypertension (see Appendix 1 for MEDLINE searches). The search was developed in MEDLINE and adapted for subsequent databases. We searched MEDLINE, EMBASE, CINAHL, Web of Science, PsycINFO and the Cochrane library from their inception to September 2017. We identified further potentially relevant articles from forward (via Google Scholar) and backward (reference list of paper) citation tracking of included studies, applying the same inclusion criteria.

### 2.2 Eligibility criteria

Following Cochrane Effective Practice and Organisation of Care (EPOC) guidance,^23^ we included randomized controlled trials (RCTs), nonrandomized controlled trials, controlled before‐after studies and interrupted time series studies. We included published studies reporting on interventions supporting shared decision making for adults (>18) with hypertension. Eligible comparator interventions were control or any other interventions. Interventions could be delivered in any health‐care setting, either before or during consultations with any health‐care professionals. We included studies describing interventions that supported shared decision making by supporting one of the two following processes of shared decision making: supporting a patient's consideration of their options in relation to a health‐care choice; or supporting a patient to consider their values and preferences in relation to a health‐care choice. We included studies in which only a proportion of participants were hypertensive, if study outcomes were reported separately for the hypertensive group. We excluded studies reporting interventions unrelated to health‐care decisions, for example, purely educational interventions that aimed to increase hypertension knowledge without reference to health‐care choices faced by the patient. We excluded interventions that aimed to increase the involvement of patients in their own care generally, but not in health‐care decisions specifically. To develop an understanding of how interventions to support shared decision making were evaluated, we included studies regardless of the outcomes assessed. No date or language restrictions were applied.

### 2.3 Reference management and study selection

EndNote X7.7 and Access 2013 were used to manage the references. Duplicates were removed from the EndNote file. Titles and abstracts, and subsequently full texts, were screened independently by two reviewers (RJ, BP or AH); disagreements were resolved by discussion with reference to a third reviewer where necessary (KT, GF and HC). If there was insufficient detail on potentially relevant studies within the report abstract, it was screened as full text. Reasons for exclusions of full‐text reports were documented.

We scrutinized the text and reference lists of relevant systematic reviews for potentially eligible studies. Conference abstracts and relevant study protocols were followed up either by contact with the author where possible or by searching for subsequent publications in PubMed.

### 2.4 Data extraction and risk of bias

Data were extracted into a custom‐designed table which had been previously piloted by one reviewer (RJ). All data were extracted by one reviewer and checked by a second. Data were extracted on study type, setting, participants, interventions, controls, type of decision supported and outcome measures. Our prespecified primary outcome was any measure of shared decision making. Consistent with our objective of documenting what outcomes have been used to evaluate interventions to support shared decision making, all other reported outcomes were extracted as secondary outcomes. We extracted estimated effect sizes with 95% confidence intervals for each outcome assessed, using odds ratios for binary variables and mean differences for continuous variables. Risk of bias was assessed independently by two reviewers using the Cochrane EPOC risk of bias tool^23^; disagreements were resolved by discussion. Risk of bias in some domains varied with the type of outcome measure; risk of bias grouped by type of outcome is presented in Figure 2.

### 2.5 Data synthesis

For data pooling, where outcomes were assessed using different measures, we planned to calculate standardized mean differences (SMDs). Meta‐analysis was planned if there were at least three studies with comparable interventions and outcomes at low risk of bias. If meta‐analysis was appropriate, we planned to assess heterogeneity amongst studies using the *I* ^2^ statistic. Analyses were carried out using Stata version 14.1.^24^

As meta‐analysis did not prove possible, we present a narrative synthesis of the studies.^25^ The included studies are summarized in the text, in a table of study characteristics and in a risk of bias summary table. The outcomes reported by included studies, grouped by type of intervention, are reported in Figure 3. Outcomes reported by at least three of the included studies are compared across the studies in forest plots and in the text.

## 3 RESULTS

**Figure 1:**
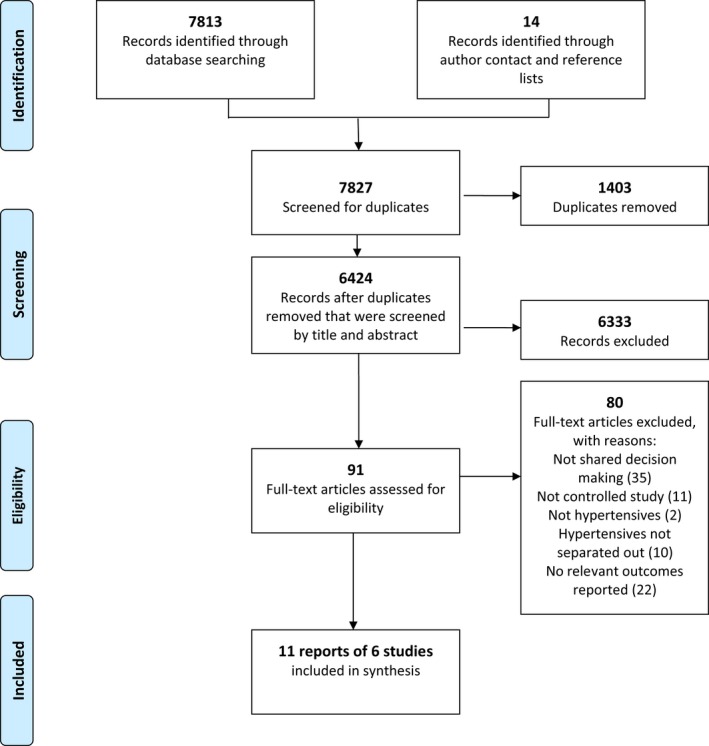
PRISMA flow diagram

Searches were run in December 2014 and updated in September 2017. A total of 6424 unique articles were screened, of which 91 full‐text articles were assessed, and 11 reports of 6 studies were included in the review (Figure 1).^26^, ^27^, ^28^, ^29^, ^30^, ^31^, ^32^, ^33^, ^34^

### 3.1 Included studies

Eleven papers were published from six studies, all based in primary care (Table 1). Five studies reported randomized controlled trials,^26^, ^27^, ^29^, ^30^ of which two were cluster randomized.^29^, ^30^ The remaining study was a nonrandomized controlled study.^28^

**Table 1:** Characteristics of included studies

Study, year, country Design N = randomized Setting/recruitment	Baseline characteristics of participants:	Intervention(s) n = number randomized	Control n = number randomized
Watkins 1987 United Kingdom RCT N = 565 Primary care Patients dispensed antihypertensives by pharmacy OR Patients on GP hypertension disease register	Only whole sample data reported Age: “almost 2/3 were 55‐64” % female: 59% Ethnicity: 27% non‐Caucasian Hypertension status: “very similar with respect to diastolic blood pressure prior to the start of the study” Of 75% (313) having BP recorded in 6 mo prior to the study, 147 (47%) had DBP of at least 95 mmHg	n = 204 participants analysed; numbers randomized not reported by intervention group Information and medical record booklet: Mailed booklet with information on hypertension including treatment options, with the aim of providing an opportunity for the general practitioner and patient to set the objectives of management together and to share information on how well these had been obtained.	n = 210 participants analysed; numbers randomized not reported by intervention group control intervention not further specified
Montgomery 2003 Emmett 2005 (3‐y follow‐up) United Kingdom 2 × 2 factorial RCT (patients randomized) 4 groups*: 1. Decision analysis; 2. Decision analysis + video/leaflet; 3. Video/leaflet; 4. Usual care N = 217 Primary care Age 30‐80 not currently taking antihypertensives, BP sustained at a level where GP would normally discuss initiation of pharmacological therapy.	Age (SD): Intervention: Decision analysis alone 59 (9), Decision analysis + video/leaflet 57 (11) Control: Usual care 58 (11), Video/leaflet 60 (10) % female: Intervention: Decision analysis alone 46% Decision analysis + video/leaflet 49% Control: Usual care 49%, Video/leaflet 47% Ethnicity: not reported Hypertension status: Mean SBP/DBP in mmHg (SD) Intervention: Decision analysis alone 167 (11)/99 (6) Decision analysis + video/leaflet 170 (14)/98 (8) Control: Usual care 169 (13)/100 (9) Video/leaflet 166 (14)/97 (8)	n = 103, of which: 52 received decision analysis alone, 51 received decision analysis + video/leaflet Decision analysis session (1 h with researcher), in which patient participant's values regarding treatment outcomes are combined with individual cardiovascular risk information to create a decision tree to support decision making. Results of the decision analysis are presented as a paper summary Video/leaflet: Factual information including about BP, self‐help measures and BP medication	n = 114, of which: 55 received video/leaflet in addition to usual care, and 59 received usual care Usual care—not further specified Video/leaflet: Factual information including about BP, self‐help measures and BP medication
Deinzer 2009 Deinzer 2006 Germany Nonrandomized controlled N = 86 Primary care *Patients:* BP>/= 135/85 mmHg, excluding those with severe hypertension (BP >/= 160/100 mmHg), poor control, established cardiovascular disease or diabetes mellitus *GPs* (not characterized)	Intervention, control *Age (SD):* 60.9 (10.1), 61.1(9.3) Female (%): 67.5%, 65% Ethnicity: Not specified Hypertension status: Mean systolic blood pressure mmHg (SD): 145.4 (11.7), 144.9 (11.1) Mean diastolic blood pressure mmHg (SD): 86.6 (8.2), 86.1 (9.1)	n = 40 Training programme for GPs “to develop communication skills necessary to practice shared decision making” Regular supervision of trained physicians Regular consultations between trained physicians and patients to make decisions on further treatment (at 1, 3, 6 and 12 mo) Hypertension education module for patients	n = 46 Hypertension education module for patients
Cooper 2011 Cooper 2009 (protocol paper) USA 2 × 2 factorial RCT N = 279 patients N = 50 physicians 4 groups: 1. Physician intensive intervention/patient intensive intervention; 2. Physician minimal intervention/patient intensive intervention; 3. Physician intensive intervention/patient minimal intervention; 4. Physician and patient minimal intervention (serves as reference group for comparisons) Patients: Adults (18+ y) with hypertension *Physicians:* General internists/family physicians seeing patients in community‐based primary care sites	Patient participants Physician intensive/patient intensive: Age (SD): 59.7 (11.9) Female (%): 65.1 Ethnicity (%): African American 62.6% Asian 2.4% American Indian 0% White 34.9% REALM >/= 9th Grade: 59.8% Physician minimal/patient minimal Age (SD): 62.4 (12.1) Female (%): 61.8 Ethnicity (%): African American 58.2% Asian 0% American Indian 1.8% White 40% REALM >/= 9th Grade: 70.9%	n (patients) = 224 Intervention groups: Physician intensive/patient intensive, n = 83 Physician minimal/patient intensive, n = 57 Physician intensive/patient minimal, n = 84 Patient intensive intervention: Previsit coaching, by community health workers (CHWs) to support patient participation. CHWs supported patients to identify changes they wanted to make to their interactions with their physicians, including practising asking questions and stating preferences. Stage 1: 20‐min previsit coaching session prior to index visit with physician; 10‐min debriefing after the visit. Stage 2: (i) 5 × 10‐15‐min phone calls over 12 mo; telephone support between these times (ii) Bimonthly photonovel depicting patients and physicians dealing with daily challenges of hypertension management (iii) Monthly newsletter including information about living with hypertension Physician intensive intervention: Communication skill training programme: Videotaped consultation between physician and simulated patient (African American hypertensive man) prior to the study randomization. Physician receives CD‐ROM on which the videotaped consultation is recorded and coded (using Roter interaction analysis system), with individualized feedback on communication skills relevant to increasing patient engagement, activation, empowerment and adherence. Five specific behaviours targeted: 1. Elicit full spectrum of the patient concerns; 2. Probe pts hypertension knowledge and beliefs; 3. Monitor adherence and identify barriers; 4. Assess adherence‐related lifestyle and psychosocial issues; 5. Elicit commitment to the therapeutic plan An accompanying workbook includes exercises for the physician to complete. Estimated time to complete workbook: 2 h Physicians receive a copy of the JNC‐VII hypertension treatment guidelines at baseline and a monthly newsletter with study updates/recent evidence updates	n (patients) = 55 The “Physician minimal/patient minimal” serves as reference group with which changes in outcome are compared: Patient minimal intervention: Monthly newsletter including information about living with hypertension Physician minimal intervention: Videotaped consultation with a simulated patient (African American hypertensive man) prior to the study randomization; no feedback on the consultation is received Physicians receive a copy of the Joint National Committee 7th report hypertension treatment guidelines at baseline and a monthly newsletter with study updates/recent evidence updates
Tinsel 2013 Germany Tinsel 2012 (protocol paper) Germany Cluster RCT (randomization at practice level) Primary care N (GP practices) = 36 N (patients) = 1120 Practices: Located in south‐west Germany; offering the full spectrum of family doctor's health‐care services; not participating in another study of shared decision‐making implementation Patients: Prescribed regular antihypertensive medications, who *either* have poorly controlled BP (24 h mean >130/80) *or* controlled BP with cardiovascular comorbidity	Intervention, control Age (SD): 63.8 (12.1), 65.0 (± 12.4) Female (%): 53.3%, 55.3% Ethnicity: Not reported Hypertension status: Mean SBP in mmHg (SD) 128.9 (12.5), 127.0 (11.8) Mean DBP in mmHg (SD) 79.2 (9.5), 76.8 (9.1)	17 GP practices n (patients) = 552 Training programme for GPs. Training was delivered over two or three sessions of 3 h each and included education about hypertension, principles of risk communication, implementation of shared decision making, use of motivational interviewing, the use of a decision aid listing options to lower cardiovascular risk and role‐playing of case vignettes Cardiovascular risk table “including elements of shared decision making” Patient information flyers for GPs to distribute Six‐monthly ambulatory blood pressure measurements and GP consultation at which blood pressure management was discussed and outcomes measured	19 GP practices n (patients) = 568 Usual care Six‐monthly ambulatory blood pressure measurements and GP consultation at which blood pressure management was discussed and outcomes measured
Denig 2014 Denig 2012 (protocol paper) The Netherlands Cluster RCT with 2 × 2 factorial design with a control group (randomization at practice level (computer version or printed version), and subsequently at patient level [short version, extended version, or control]) General practice N (practices) = 18 N (patients) = 344 Practices: General practices in the north Netherlands Patients: Patients with diabetes under age 65 when diagnosed, excluding those with recent cardiovascular events Considered eligible for BP treatment intervention when SBP>= 140	Intervention, control Age (SD): 61.8 (8.5), 61.5 (8.5) Female (%): 42%, 26% Ethnicity: not reported Low educational attainment: 40%, 38% Hypertension status: Uncontrolled SBP >=140 mmHg (%) 50%, 42%	n (patients) = 225 Prior to the study, health‐care professional received training course in motivational interviewing and risk communication Decision aid for use before consultation (patient) and during consultation (with health‐care professional) including tailored information on risks and treatment options for multiple risk factors (Hba1c, SBP, LDL and smoking), focusing on shared goal setting and decision making Two forms of the decision aid were assessed using the factorial design: SHORT version presenting risk of myocardial infarction only, or EXTENDED version presenting additional outcomes	n (patients) = 119 Usual care Components of intervention: Prior to the study, health‐care professionals received training course in motivational interviewing and risk communication

BP, blood pressure; CHW, community health worker; DA, decision analysis; DBP, diastolic blood pressure; GP, general practitioner; Hba1c, glycated haemoglobin; JNC‐VII, The Seventh Report of the Joint National Committee; LDL, low‐density lipoprotein; RCT, randomized controlled trial; REALM, rapid estimate of adult literacy in medicine; SBP, systolic blood pressure; SD, standard deviation.

### 3.2 Profile of patients

The range of mean age of study participants was 58.5‐64.5 years, and the range of female participants was 32.5%‐66.0%. In five studies, all recruited patients had hypertension.^26^, ^27^, ^28^, ^29^, ^34^ In the remaining study,^30^ only a proportion of participants were hypertensive, although all had raised cardiovascular risk. Only results relating to the hypertensive patients within this study are included in this review.^30^

### 3.3 Profile of interventions

The interventions were heterogeneous in their content and often multicomponent (Table 1). Intervention components included training interventions for clinicians,^28^, ^29^, ^34^ coaching for patients, decision aids and written materials for patients.^26^, ^34^ Tinsel and colleagues^29^, ^32^ evaluated a shared decision‐making training programme for general practitioners, to understand whether it increased patients’ perceived participation, optimized blood pressure values, enhanced patient knowledge of hypertension and improved adherence. Deinzer and colleagues^28^, ^35^ evaluated a shared decision‐making training intervention for general practitioners,^28^ testing the hypothesis that shared decision making would lead to more effective lowering of hypertension. In the study by Cooper and colleagues,^34^, ^36^ a communication skill training intervention for physicians and a coaching intervention for patients were evaluated, separately and in combination with each other, for their impact on patient‐physician communication and care processes, patient adherence to medication and lifestyle recommendations, and blood pressure control. In two studies, the main intervention component was a decision aid.^27^, ^28^, ^29^, ^30^ In the first of these, Denig and colleagues^30^, ^31^, ^32^, ^33^ set out to support interactions between patients and health‐care providers using a decision aid focusing on shared goal setting and decision making for patients with diabetes considering their treatment options, including for management of hypertension. In the second study, Montgomery and colleagues^27^, ^31^ set out to evaluate the effect of decision analysis as an aid to patient decision making for newly diagnosed hypertension on decision quality, treatment choices, clinical outcomes, and treatment and consulting behaviour.^31^ In the final study,^26^ the intervention was a leaflet distributed to patients with hypertension and hypothesized to lead to greater involvement of patients in their health‐care choices, with the potential for improving on blood pressure control.

In four studies,^26^, ^27^, ^28^, ^29^, ^30^, ^31^, ^32^, ^33^, ^34^ interventions supported the involvement of patients with established hypertension, without specifying which treatment choices were being supported. In one study,^27^ the decision supported was whether to commence antihypertensives in newly diagnosed hypertensive patients. The intervention was an approximately hour‐long session of decision analysis which took place outside of the clinical encounter. One intervention aimed to support shared decision making in consultations where multiple treatment options to lower cardiovascular risk were being considered, including decisions about commencing antihypertensive therapy.^29^

### 3.4 Risk of bias

**Figure 2:**
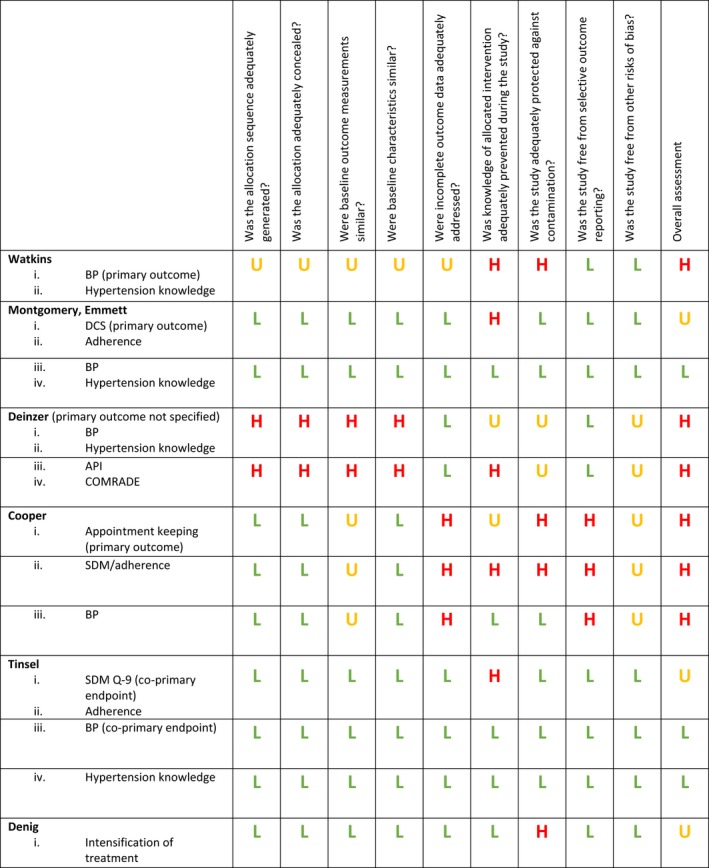
Risk of bias of primary studies. Effective Practice and Organisation of Care (EPOC) risk of bias assessment of included studies, by outcome grouping, for outcomes reported in at least three studies (Except Denig, where risk of bias is reported for the single outcome extracted for this review). BP, blood pressure; DCS, Decisional Conflict Scale; API, Autonomy Preference Index; SDM, shared decision making; SDM‐Q‐9, 9‐item Shared Decision Making Questionnaire

**Figure 3:**
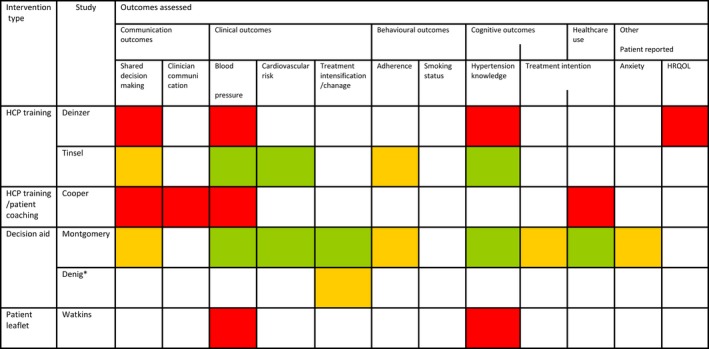
Outcomes reported in included studies, by intervention type and risk of bias. RED = high risk of bias; ORANGE = uncertain risk of bias; GREEN = low risk of bias; HCP = health‐care professional. *Outcomes reported for the study by Denig are only those reported for the hypertensive subgroup within the study

Risk of bias assessment is reported in Figures 2 and 3. One nonrandomized controlled study was included in the review and was at high risk of bias for most domains. Two of the RCTs were at uncertain or high risk of bias for the majority of domains.^26^, ^34^ Three RCTs were at low risk for most domains.^27^, ^28^, ^29^, ^30^ However, the two RCTs reporting shared decision making were at uncertain risk of bias for this outcome because of the impossibility of blinding for, as well as the subjectivity of, this outcome.

### 3.5 Outcomes

The included studies assessed a range of outcome measures. Outcomes reported, by intervention type and risk of bias, are shown in Figure 3. Four studies reported a measure of shared decision making.^27^, ^28^, ^32^, ^34^ Clinical outcomes reported were as follows: blood pressure (five studies),^26^, ^28^, ^29^, ^31^, ^34^ hypertension treatment (two studies),^30^, ^31^ cardiovascular risk (two studies),^31^ diagnosis of diabetes, left ventricular hypertrophy and lipid profile (all reported in a single study).^31^ Behavioural utcomes were medication adherence (three studies),^29^, ^31^, ^34^ smoking status (one study)^31^ and intention to start treatment (one study).^31^ Anxiety was the only psychological outcome reported (one study).^27^ Cognitive outcomes were hypertension knowledge (four studies)^26^, ^29^ and intention to start treatment (one study).^27^ Only one study reported a measure of health‐care use.^30^ Other outcomes included health‐related quality of life (one study)^28^ and clinician communication (one study).^33^

Here, we discuss our primary outcome (shared decision making), and the outcomes reported in at least three of the included studies (blood pressure, hypertension knowledge and medication adherence). The decision to limit our discussion to the most commonly reported one was a post hoc decision, as detailed reporting of all of the outcomes reported was not practical. All outcomes are reported in Table 2. None of the outcomes met our prespecified criteria for meta‐analysis of at least three studies with comparable interventions and outcomes at low risk of bias; therefore, we did not pool data for any outcome.

**Table 2:** Study outcomes

Study	Shared decision‐making measure used	Shared decision making Intervention vs Control	Outcomes: Intervention (I), Control (C) Blood pressure Hypertension knowledge Adherence	Other outcomes
Watkins	Not measured	Not measured	Blood pressure: At 1 y SBP (mmHg) Adjusted mean (SE): I = 149.8 (2.6), C = 149.2 (2.6), *P* (for test of difference between two comparison groups) <0.001 DBP (mmHg): I = 95.3 (1.7), C = 94.9 (1.7) NS *P* (for test of difference between two comparison groups) <0.001 Hypertension knowledge Knowledge score at 1 y: Adjusted mean (SE): I = 25.95 (0.21), C = 25.08 (0.21) *P* (for test of difference between two comparison groups) <0.001	
Montgomery (2003) Emmett (2005) (3‐y follow‐up)	Decisional Conflict Scale (DCS) A 16‐item patient self‐report scale. The 16‐item scale comprises five subscales, assessing the quality of the decision, and the extent to which a patient reports being informed, clear about their values, supported and certain about their choice.	Decisional conflict scale (DCS) score Primary follow‐up (mean of 14 days after intervention) Total DCS score Decision analysis vs no decision analysis: Adjusted difference in means, (95% CI), *P* = −9.4 (95% CI −13.0 to −5.8), *P* < 0.001 Decision analysis subscales: Adjusted difference in means (95% CI) −5.4 (95% CI −10.6 to −0.2) for uncertainty subscale −15.7 (95% CI −20.2 to −11.2) for uninformed subscale −13.1 (−18.0 to −8.1) for unclear values subscale −8.7 (−12.8 to −4.7) for the unsupported subscale −1.7 (−6.0 to 2.5) for the decision quality subscale	Antihypertensive treatment: At 3 mo: Prescription of blood pressure‐lowering medication: Decision analysis vs no decision analysis OR (adjusted) 1.13 (95% CI 0.59, 2.19) *P* = 0.71 Blood pressure: At 3 y: (mean follow‐up 2.8 y, range 2.2‐3.4 y) Decision analysis vs no decision analysis: Mean SBP in mmHg (SD): 149 (14), 147 (15) Adjusted difference (95% CI): 0.94 (−3.2 to 5.1) *P* = 0.65 DBP(mmHg): 85 (8), 85 (10) Adjusted difference (95% CI): −0.76 (−3.1 to 1.6) *P* = 0.53 Hypertension knowledge: At primary follow‐up (mean 14 d after randomization): % of answers correct, mean (SD) 73 (15), 67 (16) Difference (95% CI) 5 (2‐9), *P* = 0.003 Change, from primary to 3‐mo follow‐up, in % of answers correct, mean (SD) −1.9 (10.6), −1.2 (16.6) Difference (95% CI) −1.2 (−5.1 to 2.7), *P* = 0.53 Adherence Proportion reporting taking all their medication 91% overall 90% decision analysis OR = 1.56, 95% CI 0.49‐4.96	Emotional state anxiety (range 20‐80): At primary follow‐up (mean 14 days after randomization), mean (SD) Decision analysis 34.8 (10.3), No decision analysis 36.8 (13.8) Adjusted difference (95% CI): Decision analysis ‐no decision analysis = −2.8 (−5.6 to 0.1), *P* = 0.055 Change from primary to 3‐mo follow‐up mean (SD) Decision analysis −0.3 (10.9), No decision analysis −2.2 (11.10 Adjusted difference (95% CI) Decision analysis ‐ no decision analysis = 2.4 (−1.2 to 6.0) *P* = 0.19 intention to start antihypertensive treatment, after decision analysis session (mean 14 days after randomization): yes vs unsure, Decision analysis vs no decision analysis RR (CI) 1.19 (0.59‐2.4) No vs unsure, RR (CI) Decision analysis vs no decision analysis 3.15 (0.91‐10.98) Proportion prescribed antihypertensives Decision analysis vs no decision analysis OR (adjusted) 0.93 (95% CI 0.46‐1.96) *P* = 0.85 Clinical: Decision analysis vs no decision analysis Mean 10‐y cardiovascular risk (SD) 22 (11), 23 (12) Adjusted difference (95% CI) (−0.02 to 0.05) *P* = 0.54 Mean consultations per year (SD) 4.5 (2.5), 4.7 (2.1) Adjusted difference (98% CI) 0 (−0.61 to 0.6) *P* = 0.90 Mean consultations in which change to medication was made (SD) 3.1 (3.4), 3.4 (2.0) Adjusted difference (95% CI) −0.2 (−1.0 to 0.6) *P* = 0.61
Deinzer	Autonomy Preference Index (API) A patient self‐report measure of preference for participation in decision making and for information Modified COMRADE scale A 20‐item patient report scale measuring satisfaction with communication and confidence in decision made	Autonomy Preference Index (API) At 1 y “showed no difference between the SDM and control group at baseline (*P* = 0.60) and did not change after 1 y (*P* = 0.83)” (no figures reported) Modified COMRADE scale At 1 y “Both groups showed an increase in SDM” (no figures reported)	Blood pressure: Blood pressure at 1 y Unadjusted mean change from baseline (SD) SBP in mmHg: −9.26 (10.2), −6 (11.8) *P* = 0.24 DBP (mmHg): −5.33 (9.5), −3.0 (8.3) *P* = 0.19 Hypertension knowledge “After 1 y both groups showed similar levels of knowledge “(no figures given)	Health‐related quality of life No figures are given: “There were no differences between the 2 groups concerning health‐related quality of life measured with the 8 scales of SF‐36”
Cooper	Physicians’ Participatory Decision‐Making Style (PDM) This patient report measure is an aggregate score of three items (each scored 0 to 5): (i) If there were a choice between treatments, how often would this doctor ask you to help make the decision? (ii) How often does this doctor give you some control over your treatment? 3) How often does this doctor ask you to take some of the responsibility for your treatment? Higher scores reflect more participatory visits Patients’ Perceived Involvement in Care Scale (PICS) Patient self‐report measure with 3 subscales (doctor facilitation of patient involvement, information exchange between patient and physicians and patient participation in medical decision making) each scored from 1 to 5, with higher scores reflecting more involvement in care	Patient rating of clinician's participatory decision‐making style (PDM) At 12 mo Change from baseline (coefficient and 95% CI from mixed‐effects regression controlling for nesting within physician) Physician + patient intensive 6.2 (−0.5, 12.9) Physician minimal/patient intensive 3.2 (−4.8, 11.3) 13 Physician intensive/patient minimal 3.1 (−3.9, 10.2) Physician + patient minimal −5.2 (−13.0, 2.5) *P* values for the comparison of the change in PDM at 1 y between each intervention group and the reference group (physician and patient minimal): physician intensive/patient intensive group *P* = 0.03; physician minimal/patient intensive group *P* = 0.13; physician intensive/patient minimal *P* = 0.12 Patients’ Perceived Involvement in Care Scale (PICS) subscales At 12 mo Change from baseline (coefficient and 95% CI from mixed‐effects regression controlling for nesting within physician) *Subscale: doctor facilitation* Physician + patient intensive 0.22 (0.08, 0.56) Physician minimal/patient intensive 0.12 (−0.15, 0.39) Physician intensive/patient minimal 0.09 (−0.14, 0.33) Physician + patient minimal −0.17 (−0.43,0.09) Subscale: information exchange Physician + patient intensive 0.32 (0.08, 0.56) Physician minimal/patient intensive 0.16 (−0.14, 0.45) Physician intensive/patient minimal 0.13 (−0.13, 0.38) Physician + patient minimal −0.22 (−0.51, 0.07) Subscale: patient decision making Physician + patient intensive 0.21 (−0.03, 0.44) Physician minimal/patient intensive 0.07 (−0.23, 0.36) Physician intensive/patient minimal 0.16 (−0.10, 0.41) Physician + patient minimal −0.13 (−0.42, 0.16) *P* values for the comparison of the change in PICS subscales at one year between each intervention group and the reference group (physician and patient minimal): Subscale: doctor facilitation Physician intensive/patient intensive group *P* = 0.03 physician minimal/patient intensive group *P* = 0.11 physician intensive/patient minimal *P* = 0.14 Subscale: information exchange Physician + patient intensive *P* = 0.005 Physician minimal/patient intensive *P* = 0.08 Physician intensive/patient minimal *P* = 0.08 Subscale: patient decision making Physician + patient intensive *P* = 0.08 Physician minimal/patient intensive *P* = 0.35 Physician intensive/patient minimal *P* = 0.14	Blood pressure At 12 mo: Change from baseline (coefficient and 95% CI from mixed‐effects regression controlling for nesting within physician) SBP in mmHg: Physician + patient intensive −2.8 (−9.5, 2.8) *P* = 0.58 Physician minimal/patient intensive −6.5 (−14.2, 1.2) *P* = 0.24 Physician intensive/patient minimal −2.3 (−9.7, 4.0) *P* = 0.65 Physician + patient minimal −0.1 (−0.75, 7.4) *P* values for the comparison of the change in SBP at 1 y between each intervention group and the reference group (physician and patient minimal): Physician + patient intensive *P* = 0.58 Physician minimal/patient intensive *P* = 0.24 Physician intensive/patient minimal *P* = 0.65 DBP in mmHg: Physician + patient intensive 0.2 (−3.7, 4.1) Physician minimal/patient intensive −0.9 (−5.4, 3.6) Physician intensive/patient minimal −1.4 (−5.1, 2.3) Physician + patient minimal 0.2 (−4.1,4.6) *P* values for the comparison of the change in DBP at 1 y between each intervention group and the reference group (physician and patient minimal): Physician + patient intensive *P* = 1.0 Physician minimal/patient intensive *P* = 0.72 Physician intensive/patient minimal *P* = 0.57 Adherence (Morisky scale): At 12 mo Change from baseline (predicted probability and 95% CI from logistic mixed‐effects regression controlling for nesting within physician) Physician + patient intensive 0.75 (0.62, 0.84) Physician minimal/patient intensive 0.80 (0.65, 0.90) Physician intensive/patient minimal 0.66 (0.53, 0.77) Physician + patient minimal 0.77 (0.63, 0.87) *P* for comparison with reference group (physician and patient minimal) Physician + patient intensive *P* = 0.75 Physician minimal/patient intensive *P* = 0.76 Physician intensive/patient minimal *P* = 0.22	% with BP controlled at 12 mo conditional probability (95% CI) from mixed‐effects regression controlling for nesting within physician Physician + patient intensive 0.53 (0.38, 0.68) Physician minimal/patient intensive 0.61 (0.43, 0.77) Physician intensive/patient minimal 0.65 (0.50, 0.78) Physician and patient minimal 0.55 (0.37,0.71) *P* for comparison of the % with BP controlled to reference group (physician and patient minimal): Physician + patient intensive *P* = 0.92 Physician minimal/patient intensive *P* = 0.58 Physician intensive/patient minimal *P* = 0.35 Patient ‐ physician communication i. Change in verbal dominance ratio (first study visit with actual patient, compared with videotaped simulated visit), by physician intervention group: Change (coefficient and 95% CI from mixed‐effects regression controlling for nesting within physician) Physician intensive group: −1.67 (−2.06, −1.28) Physician minimal group: −1.94 (−2.36, −1.53) *P* for comparison of change between physician intensive and physician minimal groups *P* = 0.35 ii. Change in patient‐centredness ratio (first study visit with actual patient, compared with videotaped simulated visit), by physician intervention group: Change (coefficient and 95% CI from mixed‐effects regression controlling for nesting within physician) Physician intensive group: −0.52 (−0.71, −0.32) Physician minimal group: −0.82 (−1/02, −0.61) *P* for comparison of change between physician intensive and physician minimal groups *P* = 0.04
Tinsel	The SDM‐Q‐9^36^ is a 9‐item self‐report scale in which the patient reports the extent to which shared decision making occurred; raw scores are transformed into a scale from 1‐100 in which higher scores indicate that patients perceive more shared decision making to occur.	Shared Decision Making Q‐9 (SDM‐Q‐9) Difference in average means change (from baseline to 18 mo) from mixed‐effects model adjusted for baseline values of outcomes: Intervention vs control 3.1182, 97.5% CI −2.3730; 8.6093, *P* = 0.2029* *not statistically significant at 2.5% level [Bonferroni correction applied due to multiple outcome measures]	Blood pressure: Difference in average means change over 1 y from T1 to T3, from mixed‐effects model adjusted for baseline values of outcomes: SBP (mmHg): +1.75 mmHg (97.5% CI [−0.189; 3.69], *P* = 0.043*) *not statistically significant at 2.5% level [Bonferonni correction applied due to multiple outcome measures] DBP (mmHg): + 0.9377 (95% CI −0.0381; 1.9134 *P* = 0.0596) Hypertension knowledge: Difference in average means change from T0 to T3 (CI), from mixed‐effects model adjusted for baseline values of outcomes: 1.3267 (95% CI −4.3272; 6.9806), *P* = 0.65454 Adherence (MARS‐D) Difference in average means change from T1 to T3 (CI) from mixed‐effects model adjusted for baseline values of outcomes: 0.670 (95% CI −0.3748; 1.7166), *P* = 0.2084	Cardiovascular risk score Difference in average means change from T1 to T3 (CI) from mixed‐effects model adjusted for baseline values of outcomes: −0.4891 (95% CI −1.4307; 0.4526), *P* = 0.3084
Denig		Not measured		Antihypertensive treatment Proportion of patients eligible for intensification of blood pressure treatment (SBP >=140 mmHg) Who had blood pressure treatment intensified OR (intervention, control), *P* value intervention: intensification in 17 (16%) control: Intensification in 8 (17%) OR 0.93 (0.37‐2.34) *P* = 0.882

(S/D) BP, systolic/diastolic) blood pressure; API, Autonomy Preference Index; CI, confidence interval; DA, decision analysis; DCS, Decisional Conflict Scale; NS, nonsignificant; OR, odds ratio; PDM, Participatory Decision‐Making Score; PICS, Patients’ Perceived Involvement in Care Scale RCT, randomized controlled trial; RR, relative risk; SD, standard deviation; SDM‐Q‐9, 9‐item Shared Decision Making Questionnaire; SE, standard error.

Unadjusted results unless otherwise stated.

#### 3.5.1 Primary outcome: shared decision making—risk of bias (Figure 2) and results (Table 2 and Figure 4)

**Figure 4:**
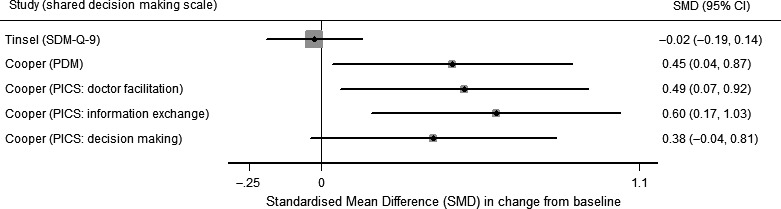
Change in shared decision making at 1 y. Forest plot of the standardized mean difference (SMD) of change from baseline for shared decision‐making scales: SDM‐Q‐9, Physicians’ Participatory Decision‐Making Style (PDM) and subscales of the Patients’ Perceived Involvement in Care Scale (PICS) [doctor facilitation, information exchange and decision making]. Tinsel: results adjusted for baseline values of outcomes. Cooper: no adjustment reported

The four studies measuring shared decision making^27^, ^29^ used different patient self‐report measures; measures are described in Table 2. Shared decision making was assessed at different times, ranging from 14 days to 18 months after the intervention. In studies in which patients received an intervention, blinding patients to treatment allocation was not possible. All studies measuring shared decision making in this review were assessed as uncertain^27^, ^29^ or high risk of bias^28^, ^34^ for this outcome, due to inadequate prevention of treatment allocation knowledge. The SMD in change from baseline for shared decision‐making measures, for studies with useable data at 12 months, is shown in Figure 3.

Tinsel and colleagues^29^ use the nine‐item Shared Decision Making Questionnaire (SDM‐Q‐9)^37^ as a coprimary outcome for the study. The mean SDM‐Q‐9 score decreased in both intervention and control groups. The difference, between intervention and control, in mean change from baseline (to approximately 18 months) was 3.1182, 97.5% CI −2.3730; 8.6093, *P* = 0.2029.

Deinzer^28^ reported two shared decision‐making measures: the Autonomy Preference Index (API)^36^ and a modified version of the COMRADE scale.^39^ In this study with a high risk of bias, the authors report that at 1 year there was no change in API from baseline in either the intervention or control group, although API scores were not reported (*P* = 0.83 for the comparison). A comparison between the COMRADE scores in the intervention and control groups was not reported.

The primary outcome in the study by Montgomery and colleagues^27^ was the Decisional Conflict Scale (DCS), a 16‐item patient self‐report scale.^40^ The DCS was measured after receipt of the intervention (mean 14 days after randomization). The adjusted difference in mean DCS score (decision analysis vs no decision analysis) was −9.4 (95% CI −13.0 to −5.8), *P* < 0.001.

Cooper and colleagues report two measures of shared decision making. The first measure is the patient‐reported Physicians’ Participatory Decision‐Making Style (PDM),^41^ and the second measure is the Patients’ Perceived Involvement in Care Scale (PICS),^42^ a measure with three subscales: doctor facilitation of patient involvement; information exchange; and patient participation in medical decision making. There were three intervention groups, physician and patient intensive, physician minimal/patient intensive and physician intensive/patient minimal, and one reference group, physician and patient minimal. For each scale and intervention group, the study reported change from baseline at 12 months and a *P*‐value from the comparison with the reference group. For all intervention groups, there was no statistical evidence of a change in PDM at 12 months. Mean PDM decreased from baseline in the reference group −5.2 (95% confidence interval −13.0, 2.5) but increased from baseline in the other intervention groups: physician intensive/patient intensive group: 6.2 (−0.5, 12.9); physician minimal/patient intensive group: 3.2 (−4.8, 11.3); and physician intensive/patient minimal: 3.1 (−3.9, 10.2). *P* values for the comparison of the change in PDM at 1 year between each intervention group and the reference group were as follows: physician intensive/patient intensive group *P* = 0.03; physician minimal/patient intensive group *P* = 0.13; and physician intensive/patient minimal *P* = 0.12. Taken together, it is uncertain whether the intervention led to a change in PDM. Similar patterns were reported for the three PICS subscales. Taken together, it is uncertain whether the intervention led to a change in PDM.

#### 3.5.2 Secondary outcomes—risk of bias (Figure 2) and results (Table 2 and Figures 5 and 6)

**Figure 5:**
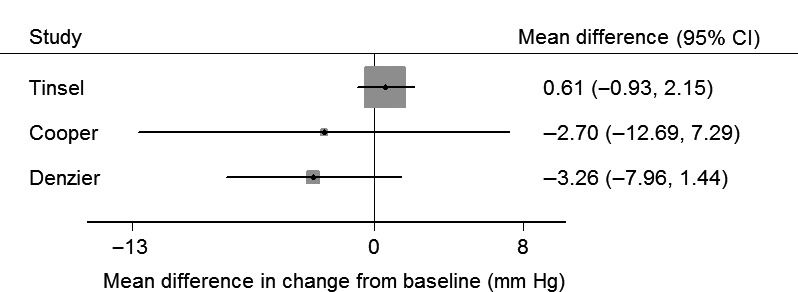
Change in systolic blood pressure at 1 y. Forest plot of the mean difference in change from baseline of systolic blood pressure (mmHg), between intervention and control. Tinsel: results adjusted for baseline values of outcomes. Cooper: no adjustment reported. Deinzer: no adjustment reported

**Figure 6:**
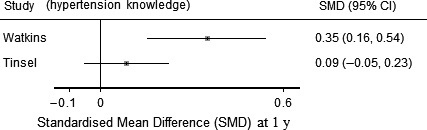
Change in hypertension knowledge at 1 y. Forest plot of the standardized mean difference (SMD) of change from baseline for hypertension knowledge. Tinsel: results adjusted for baseline values of outcomes. Cooper: no adjustment reported

Five studies evaluated the effect of the intervention on blood pressure^26^, ^28^, ^29^, ^31^ (Table 2); two studies were at low risk of bias, and three were at high risk of bias, for this outcome^26^, ^28^ (Figure 2). Blood pressure was measured at different time points (range 6 months to 3 years). Four of the five studies (two at low risk of bias) report that there was no difference between blood pressure in the intervention and control groups; in the fifth study, intervention and control were not formally compared. The mean difference in change from baseline after 1 year in three studies with useable data is shown in Figure 5.

Hypertension knowledge was assessed in four studies,^26^, ^28^, ^29^, ^31^ at different time points (range 14 days to 18 months), using different scales in each study. Results were conflicting: two studies reported that the intervention increased hypertension knowledge,^26^, ^27^ and two studies^28^, ^29^ reported that there was no statistical evidence of a difference in hypertension knowledge between intervention and control. Two studies reported comparable scales at similar time points; SMDs for these studies are reported in Figure 6.

Adherence was assessed in three studies^27^, ^29^, ^34^ at different time points (range 6 months to 3 years) and using different patient self‐report measures; two studies^29^, ^31^, ^34^ were at uncertain risk, and one study^43^ was at high risk for this outcome. In each of the three studies, there was no statistical evidence of a difference between intervention and control in patient‐reported adherence. Reporting of adherence was not comparable between the studies, and SMDs were not calculated for this outcome.

## 4 DISCUSSION

This review identified a small number of studies evaluating the effectiveness of different interventions to support shared decision making in the management of hypertension. Meta‐analysis of the included studies was not undertaken because of clinical heterogeneity (differences in interventions and outcomes) and methodological heterogeneity (differences in the risk of bias of studies). We have found that there is insufficient evidence to inform which intervention should be used to support shared decision making for hypertension in routine clinical care.

We identified six studies (five randomized controlled trials^26^, ^27^, ^29^, ^30^, ^43^ and one controlled study)^26^ evaluating interventions to support shared decision making for hypertension. The main intervention components were training for health‐care professionals (three studies),^28^, ^29^, ^34^ decision aids (two studies),^27^ patient coaching (one study)^34^ and a patient leaflet (one study).^26^ All included studies were based in primary care. No studies measuring shared decision making were at low risk of bias for this outcome. Two trials, both at uncertain risk of bias, had conflicting results: in one, a GP training intervention did not increase patient‐perceived shared decision making over 18 months,^29^ and in the second study, decision analysis reduced decisional conflict at 14 days.^27^ Of two further studies at high risk of bias,^34^ only one provided useable data^28^; in this study, it was uncertain whether an intensive intervention (clinician training and patient coaching) improved patient‐reported perceptions of clinicians’ participatory decision‐making style (PDM) or involvement in care (PICS). Four studies compared blood pressure between intervention and control^26^, ^27^, ^28^, ^29^; they reported no statistically significant difference in blood pressure at time points between 3 months and 3 years.

Of the interventions in the primary studies, only one addressed shared decision making about whether or not to initiate an antihypertensive medication, which is a key decision point in the management of hypertension. The intervention was an approximately hour‐long session of decision analysis which took place outside of the clinical encounter. This was the only study reporting increased shared decision making in the intervention group in comparison with controls, although the impossibility of blinding participants and the self‐reported nature of the outcome measure rendered the study at uncertain risk of bias. The intensity of the intervention in this study makes it unlikely to be feasible in routine health‐care settings.

Strengths of this review include the use of a comprehensive search strategy employing a range of synonyms for shared decision making. Our definition of shared decision making builds on previous research in this area; our two core components of shared decision making were the elements that appear most frequently in conceptual definitions of shared decision making^44^ and are central to the most frequently cited model of decision making.^45^ To avoid missing eligible studies, we were inclusive at the title and abstract screening stage, where intervention descriptions were often sparse. No language restrictions were used, and screening was carried out in duplicate. Uncertainties about inclusion were discussed within a multidisciplinary team of GPs/health service researchers and social scientists to ensure validity of selection. Using a narrative synthesis approach, we have been able to apply tools systematically resulting in a robust summary of the available studies, as well as highlighting where the evidence base is limited. To our knowledge, this is the first study to review interventions to support shared decision making for hypertension.

Limitations of this review include the small number of eligible studies, many of which were at uncertain or high risk of bias. The included studies described a range of interventions and evaluated a range of outcome measures, making it more challenging to summarize the data using a narrative approach. Although useful in providing an overview of the evidence available (Figure 3), this clinical heterogeneity prevented pooling of the data. An important limitation of the included studies is that measurement of shared decision‐making outcomes was biased by the lack of blinding of outcome assessment and the subjective nature of shared decision‐making outcomes. The mechanisms by which interventions might achieve their outcomes were not clearly articulated within the papers. The rationale implied in several studies is that shared decision making might enhance patient's understanding and through this compliance with antihypertensive medication. This rationale is evident in the choice of hypertension knowledge and adherence as study outcomes. Explicit acknowledgement of the mechanisms by which interventions are expected to influence outcomes including shared decision making, for example through a logic model, would be helpful in interpreting study findings.

Research in conditions other than hypertension has suggested that shared decision making has the potential to improve outcomes,^10^ increase appropriateness of care,^8^ reduce overtreatment^9^ and reduce treatment costs.^11^ Given the limitations of the studies within the review, the effects of shared decision making in hypertension remain uncertain, and none of these potential benefits can be confirmed. The interventions in several of the included studies^28^, ^29^, ^30^, ^33^ aimed to change the behaviour of clinicians in order to facilitate shared decision making. The challenges, for health professionals, in implementing shared decision making have been well described and include time constraints and the perceived lack of applicability of shared decision making to the particular clinical situation.^7^ A recent review focussing on studies measuring shared decision making and patient outcomes found that shared decision making, when perceived to be happening by patients, tended to result in improved affective‐cognitive outcomes, but that evidence was lacking for patient behavioural and health outcomes.^46^ Consistent with this review, we found that all of our included studies that measured shared decision making used a patient‐reported measure.

In the care of people with hypertension, there is a potential conflict between the aim of ensuring shared decision making occurs, and the aim of optimizing blood pressure control. Several of the included studies aimed to do both. The effect of shared decision making on clinical outcomes is important because, should it be implemented widely, it has the potential to impact on public health outcomes.^47^ For example, should the consequence of shared decision making be that fewer people take antihypertensive medication, this will increase cardiovascular events. However, the rationale for shared decision making is not to improve compliance with clinical or public health priorities, and it is to achieve a decision which is congruent with the patient's personal priorities, values and beliefs. This potential conflict was not discussed in the study reports.

## 5 CONCLUSION

Hypertension is a long‐term condition in which patients and their clinicians frequently face choices about starting or modifying hypertension treatment. Shared decision making is increasingly advocated for all health‐care choices, including those taken in the care of long‐term conditions.^5^ Decision aids continue to proliferate,^48^ and front‐line clinicians have called for more decision support interventions to help them to share decisions with patients. In this study, we have shown that there is little evidence to guide a choice of interventions to support shared decision making for hypertension.

There is insufficient evidence to recommend how to support shared decision making for patients with hypertension in routine clinical care. Further studies are needed to develop and test interventions able to support patients to share decisions with their clinicians and which can be incorporated into routine care. Future research should make explicit the underpinning theory of the intervention's mechanism of effect and should consider using observer‐rated measures of shared decision making.

## CONFLICT OF INTEREST

All authors declare that they have no conflict of interests.

## APPENDIX 1

### Medline search strategy

Database: Medline 1950 to present

1. (shared decision* or sharing decision* or informed decision* or informed choice* or joint decision*).mp
2. ((share* or sharing or informed or participat* or support*) adj2 (decision* or decid* or choice*)).ti,ab
3. Or/1‐2
4. Decision making/or Decision support techniques/or Decision Support Systems, Clinical/or Choice Behaviour/
5. ((decision* or choice*) adj2 (making or support* or behaviour* or aid*))ti,ab
6. Or/4‐5
7. ((patient* or consumer*) adj4 (involv* or participat* or enable* or empower* or engage* or partner*)).ti,ab
8. Professional‐patient relations/
9. Nurse/or physician/or (nurse*or physician* or clinician* or doctor* or general practitioner* or gp* or health care professional* or healthcare professional* or health care provider* or healthcare provider* or resident*).ti,ab
10. Patients/or (patient* or consumer* or people* or individual*).ti,ab
11. 9 and 10
12. 11 or 8
13. Patient participation/
14. 3 or (6 and 7) or (6 and 12) or 13
15. Exp hypertension/
16. (hypertens* or antihypertens*).tw
17. ((high or elevat* or rais*) adj2 blood pressure).tw
18. Or/15‐17
19. 14 and 18( )The following steps were added to the search strategy for the search update in September 2017 (the initial search strategies were used to concurrently identify studies for a qualitative synthesis):
20. intervention?.ti. or (intervention? adj6 (clinician? or collaborat$ or community or complex or DESIGN$ or doctor? or educational or family doctor? or family physician? or family practitioner? or financial or GP or general practice? or hospital? or impact? or improv$ or individuali?e? or individuali?ing or interdisciplin$ or multifacet$ or multi‐facet$ or multimodal$ or multi‐modal$ or personali?e? or personali?ing or pharmacies or pharmacist? or pharmacy or physician? or practitioner? or prescribe$ or prescription? or primary care or professional$ or provider? or regulatory or regulatory or tailor$ or target$ or team$ or usual care)).ab.
21. (pre‐intervention? or preintervention? or pre intervention? or post‐intervention? or postintervention? or post intervention?).ti,ab.
22. (hospital$ or patient?).hw. and (study or studies or care or health$ or practitioner? or provider? or physician? or nurse? or nursing or doctor?).ti,hw.
23. Demonstration project?.ti,ab.
24. (pre‐post or pre test$ or pretest$ or posttest$ or post test$ or (pre adj5 post)).ti,ab.
25. (pre‐workshop or post‐workshop or (before adj3 workshop) or (after adj3 workshop)).ti,ab.
26. Trial.ti. or ((study adj3 aim?) or our study).ab.
27. (before adj10 (after or during)).ti,ab.
28. (quasi‐experiment$ or quasiexperiment$ or quasi random$ or quasirandom$ or quasi control$ or quasicontrol$ or ((quasi$ or experimental) adj3 (method$ or study or trial or design$))).ti,ab,hw.
29. (time series adj2 interrupt$).ti,ab,hw.
30. (time points adj3 (over or multiple or 3 or 4 or 5 or 6 or 7 or 8 or 9 or 10 or 11 or 12 or month$ or hour? or day? or more than)).ab.
31. Pilot.ti.
32. Pilot projects/
33. (clinical trial or controlled clinical trial or multicentre study or randomized controlled trial).pt.
34. (multicentre or multicenter or multi‐centre or multi‐center).ti.
35. Random$.ti,ab. or controlled.ti.
36. (control adj3 (area or cohort? or compare? or condition or design or group? or intervention? or participant? or study)).ab.
37. (control year? or experimental year? or (control period? or experimental period?)).ti,ab.
38. Evaluation studies as topic/or prospective studies/or retrospective studies/or clinical trials as topic/
39. (Utili?ation or programme or programmes).ti.
40. (during adj5 period).ti,ab.
41. ((strategy or strategies) adj2 (improv$ or education$)).ti,ab.
42. (purpose adj3 study).ab.
43. placebo.ab.
44. “comment on”.cm. or review.pt. or (review not peer review$).ti.
45. (rat or rats or cow or cows or chicken? or horse or horses or mice or mouse or bovine or animal?).ti,hw. or veterinary$.ti,ab,hw.
46. exp animals/not humans.sh.
47. OR/20 ‐ 43
48. OR/44‐46
49. 47 NOT 48
